# A Cross-Sectional Study of the Relationship between Serum Creatine Kinase and Liver Biochemistry in Patients with Rhabdomyolysis

**DOI:** 10.3390/jcm9010081

**Published:** 2019-12-28

**Authors:** Andy K. H. Lim, Chitherangee Arumugananthan, Corinne Lau Hing Yim, Lucy J. Jellie, Elena W. W. Wong, Ralph K. Junckerstorff

**Affiliations:** 1 General Medicine, Monash Health, Clayton, Victoria 3168, Australia; chitherangee.arumugananthan@monashhealth.org (C.A.); corinne.yim@monashhealth.org (C.L.H.Y.); lucy.jellie@monashhealth.org (L.J.J.); elena.wong@monashhealth.org (E.W.W.W.); ralph.junckerstorff@monashhealth.org (R.K.J.); 2 Department of Medicine, Monash University, Clayton, Victoria 3168, Australia

**Keywords:** rhabdomyolysis, muscle injury, liver function tests, creatine kinase, aminotransferases, alanine aminotransferase

## Abstract

Abnormal liver function tests are commonly observed with rhabdomyolysis, but the nature of this association is not fully defined. This study aims to determine the functional relationship between serum creatine kinase, as a marker of rhabdomyolysis severity, and liver biochemistry. We used linear regression to model the relationship between liver biochemistry and peak serum creatine kinase. A total of 528 patients with a median age of 74 years were included. The distribution of creatine kinase, bilirubin, alkaline phosphatase, alanine aminotransferase, and γ-glutamyl transferase were significantly skewed, and these variables were log-transformed prior to regression. There was a positive linear correlation between log-alanine aminotransferase and log-creatine kinase. In the multiple regression analysis, log-creatine kinase, age, acute kidney injury stage, and chronic liver disease were independently associated with log-alanine aminotransferase. This model explained 46% of the variance of log-alanine aminotransferase. We found no correlation between the log-creatine kinase and the log-bilirubin, log-alkaline phosphatase, or log-γ-glutamyl transferase. Serum alanine aminotransferase was not associated with inpatient mortality but a higher creatine kinase-alanine aminotransferase ratio was associated with lower odds of mortality. In conclusion, an isolated elevation in alanine aminotransferase can occur in rhabdomyolysis, and it may be possible to anticipate the level of increase based on the peak creatine kinase.

## 1. Introduction

Rhabdomyolysis is a syndrome of skeletal muscle injury. Severe cases may present with muscular pain and weakness, brown discoloration of urine, compartment syndrome, and disseminated intravascular coagulation. The breakdown of muscle results in the release of cellular contents into the circulation, which are chiefly responsible for the systemic complications. Excess amounts of potassium and phosphate, along with hypocalcemia from calcium sequestration, contribute to the risk of cardiac arrhythmias. Myoglobin released from muscle can cause vasoconstriction, tubular casts, and oxidative stress in the kidney, resulting in acute kidney injury (AKI). Along with myoglobin, a number of enzymes are released including creatine kinase (CK), lactate dehydrogenase, and aldolase [1]. Rhabdomyolysis is due to diverse causes that are inherited or acquired. Acquired causes are more common and include trauma, drugs and toxins, pressure injuries, strenuous exercise, thermal extremes, seizures, infections, severe electrolyte abnormalities, and limb ischemia [1].

The serum CK is used to confirm a diagnosis of rhabdomyolysis and as a marker of severity. Most studies accept a level five times above the upper limit of normal (1000 U/L) for diagnosis [1], as complications such as AKI are generally not reported at CK levels below 1000 U/L [2]. However, what is less appreciated is that aminotransferases are present in muscle, and include aspartate aminotransferase (AST) and alanine aminotransferase (ALT) [3]. AST and ALT are typically considered liver enzymes but there is observational and experimental evidence that they are transiently increased in rhabdomyolysis [3,4,5,6,7]. ALT and AST are distributed in many tissues other than the liver. ALT is more specific for the liver, where it has the highest concentration relative to other tissues such as skeletal muscle, red blood cells, kidney, brain, and heart. ALT is about 5–10 times more abundant in liver compared to muscle [8,9]. However, adults have an average of 20–30 kg of skeletal muscle mass [10], and the relatively small amounts of ALT in muscle may still result in a detectable increase in blood when large muscle groups are injured.

The detection of abnormal liver function tests with rhabdomyolysis has potential implications. It may result in unnecessary tests for liver disease as clinicians may incorrectly attribute the elevated aminotransferases to liver injury rather than muscle injury, to the extent of performing invasive tests such as liver biopsies [7]. Furthermore, failure to appreciate this association can also result in a missed diagnosis of muscle disease. The finding of abnormal aminotransferases also has significant implications for clinical trials of novel treatments and critical medicines with potential hepatoxicity, where exercise-induced muscle injury have resulted in elevated serum aminotransferases which have complicated the outcomes of some clinical studies [11,12]. Finally, there may be prognostic implications for a subset of patients with critical illness and rhabdomyolysis, where a significantly elevated ALT or AST may be associated with higher mortality [13]. Thus, it is important that further analysis of this association be undertaken. Currently, there are limited studies that have examined the nature of the relationship between liver biochemistry and CK levels in patients with severe rhabdomyolysis. Until novel biomarkers that can clearly distinguish liver from muscle injury [14] become available, an improved understanding of the functional form of this relationship may assist clinicians in their decision-making on testing for liver injury.

## 2. Methods

### 2.1. Study Aim, Design and Setting

The aim of this study was to define and quantify the relationship between the serum CK and liver function tests in patients admitted to hospital with rhabdomyolysis from diverse causes. This study was based on a patient cohort which we have previously examined for AKI outcomes [15], and the patients included in this current study represented a subset of that cohort. In brief, we used the cross-sectional biochemistry data from this rhabdomyolysis cohort to examine the association between CK and liver function tests. This study was conducted at Monash Health, a large referral center in the south-east region of the city of Melbourne, in the state of Victoria, Australia. We are the largest public health network in the state, caring for around one-quarter of Melbourne’s population.

### 2.2. Patient Selection and Exclusions

To identify eligible patients with rhabdomyolysis, we used the International Classification of Diseases (10th edition) codes for muscle injury for a five-year period (January 2013 to December 2017) at three acute centers within the network (Monash Medical Centre, Dandenong Hospital and Casey Hospital). We included adult patients (≥18 years old) with a clinical syndrome compatible with rhabdomyolysis and an elevated serum CK. Duplicated entries due to transfers within the network were excluded. We excluded patients with an inadequate CK profile, peak CK < 1000 U/L, and CK rise due to isolated myocardial injuries such as myocardial infarction and cardiac arrest. We also excluded patients who had an inadequate liver biochemistry profile (missing or unpaired with CK level), ischemic hepatitis (typical clinical setting, shock and ALT rise over 3000 U/L), or acetaminophen overdose with toxic levels requiring N-acetyl cysteine treatment.

### 2.3. Variable Definitions

Rhabdomyolysis was defined as a serum CK over 1000 U/L. The peak CK was defined as the highest CK level measured during admission. To ensure a tight chronological pairing between liver function tests and CK, we only included liver biochemistry tests which were performed concurrently or within 24 h of the CK measurement. If such a pairing was not possible, patients were excluded due to an inadequate profile. Chronic kidney disease (CKD) stage was determined by the Chronic Kidney Disease Epidemiology Collaboration estimated glomerular filtration rate (eGFR). We used the Kidney Disease: Improving Global Outcomes 2012 criteria to define AKI [16] and the method recommended by Siew et al. to determine the baseline kidney function [17]. Chronic liver disease was defined as a prior record of non-alcoholic fatty liver disease, alcoholic liver disease, chronic viral hepatitis, autoimmune hepatitis, or biliary disease such as primary biliary cirrhosis. Excessive alcohol intake was defined as consumption of more than two standard drinks daily. Illicit drug use was determined from clinical history and supplemented by toxicology analysis.

### 2.4. Potentially Hepatotoxic Medication

To identify a potentially hepatotoxic medication (PHM), we referred to the LiverTox online database. LiverTox is a collaboration between the National Institute of Diabetes and Digestive and Kidney Disease, and the National Library of Medicine [18]. A medication was only considered a PHM if it was categorized as a likelihood class A or B for drug-induced liver injury. PHMs started after peak CK were disregarded as they were not relevant to the hypothesis of the study.

### 2.5. Statistical Analysis

We performed correlation analysis with Pearson’s correlation coefficient (*r*). We used multiple linear regression to examine the association between ALT and serum CK, while adjusting for other independent variables. We performed logarithmic transformations of continuous variables with significant positively skewed distributions prior to regression analysis. Linearity of continuous independent variables was examined using fractional polynomials. From the univariable analysis, we used a forward addition approach by initially including variables explaining most of the variance in ALT. We checked potential confounders for actual confounding by its effect on the regression coefficient of serum CK. We checked the final model for statistical interactions at a 1% level of significance, and collinearity by examining the variance inflation factor. Final model diagnostics included assessment of the distribution of residuals, and assessment of outliers and influential points using residual vs. fitted and leverage plots. We used a *t*-test or ANOVA to compare the means of log-transformed variables when assessing for potential confounders. We used the Wilcoxon paired sign-rank test to compare biochemistry at different time points in evaluating the trajectory of ALT and CK. We used logistic regression to determine the association between ALT and inpatient mortality. Analyses were performed with STATA 15.1 (StataCorp, Texas, TX, USA) and *p* < 0.05 was considered statistically significant.

### 2.6. Ethics Approval and Consent

This study was approved by the Monash Health Human Research Ethics Committee (RES-19-0000-305Q), an institutional review board. Patient consent was waived as this was a retrospective study using de-identified and aggregate data for analysis.

## 3. Results

### 3.1. Patient Characteristics

**Figure 1:**
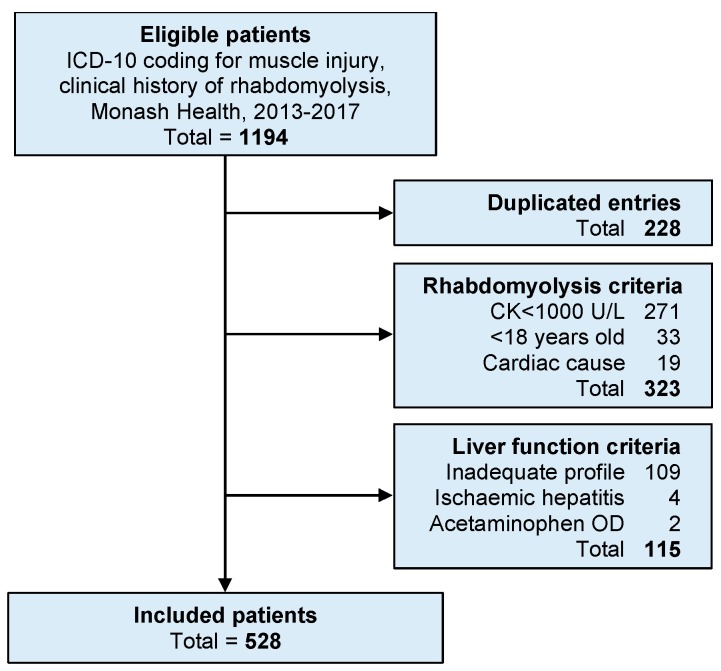
Study flow diagram. This flow chart shows the patient selection based on the International Classification of Diseases 10th revision (ICD-10) and reasons for exclusion from the study. The majority of patients were excluded for a peak creatine kinase (CK) below 1000 U/L or an inadequate liver biochemistry profile which could not be matched to the CK level.

We included 528 patients in the final analysis (Figure 1). Patient characteristics are summarized in Table 1. The major mechanism of rhabdomyolysis was pressure injury, which was implicated in 72% of patients. Most pressure injuries were due to falls and immobilization, which explains much of the older age distribution. The causes of rhabdomyolysis are shown in Appendix A Table A1. Around half of the patients had a reduced baseline eGFR and half experienced AKI. The etiology of chronic liver disease included non-alcoholic fatty liver disease (21/528), alcohol-related liver disease (17/528), chronic viral hepatitis (31/528), and others (3/528). Illicit drug use was identified in 13.1% of patients (Appendix A Table A2). In terms of PHMs, 217/528 (41.1%) were not on any PHMs, 196/528 (37.1%) were on one, 94/528 (17.8%) were on two, and 21/528 (4.0%) were on three. This included the use of statins in 188/528 (35.6%) of patients. A full categorical listing is shown in Supplementary Table S1.

### 3.2. Distribution and Trend of CK and Liver Biochemistry

The biochemistry prior to admission, at admission and at peak CK are summarized in Table 2. There were insufficient pre-admission CK and International normalized ratio (INR) readings for a meaningful aggregate. The distribution of serum CK and all liver biochemistry elements except albumin showed a significant positive skew. Serum CK continued to rise after admission in 188/528 (35.6%) of patients, resulting in an overall difference in the peak CK compared to admission CK (*z* = 13.5, *p* < 0.001). Similarly, the overall CK/ALT ratio was higher after admission than at presentation (*z* = 7.4, *p* < 0.001). On average, serum albumin dropped from baseline to admission (*n* = 126, *z* = 2.6, *p* = 0.009) and from admission to peak CK (*n* = 528, *z* = 8.8, *p* < 0.001). Bilirubin was higher on admission compared to baseline (*n* = 126, *z* = 5.6, *p* < 0.001) but was not higher than admission at the time of peak CK (*n* = 528, *z* = 0.18, *p* = 0.86). Alkaline phosphatase (ALP) was similar on admission compared to baseline (*n* = 126, *z* = 1.7, *p* = 0.09) but decreased slightly after admission to the peak CK (*n* = 528, *z* = 6.5, *p* < 0.001). GGT was not distributed differently at baseline, admission or at peak CK. Serum ALT was higher on admission compared to baseline (*n* = 126, *z* = 8.1, *p* < 0.001) and increased further after admission to peak CK (*n* = 528, *z* = 5.7, *p* < 0.001).

### 3.3. ALT at Discharge

A serum ALT was available at discharge in 43.3% of patients. For these patients, there was an overall lower ALT at discharge compared to the peak ALT, with a median decline of 11 U/L (interquartile range (IQR), 4–54 U/L). Specifically, discharge ALT was lower than peak ALT in 72% (164/229) of patients. Table 3 shows the categorical distribution of ALT at peak and discharge based on sex-specific cut-offs. Overall, 72% (165/229) of ALTs were above the reference range at the time of peak ALT, of which 28% (46/165) normalized prior to discharge.

### 3.4. Transformation of CK and Liver Biochemistry

**Figure 2:**
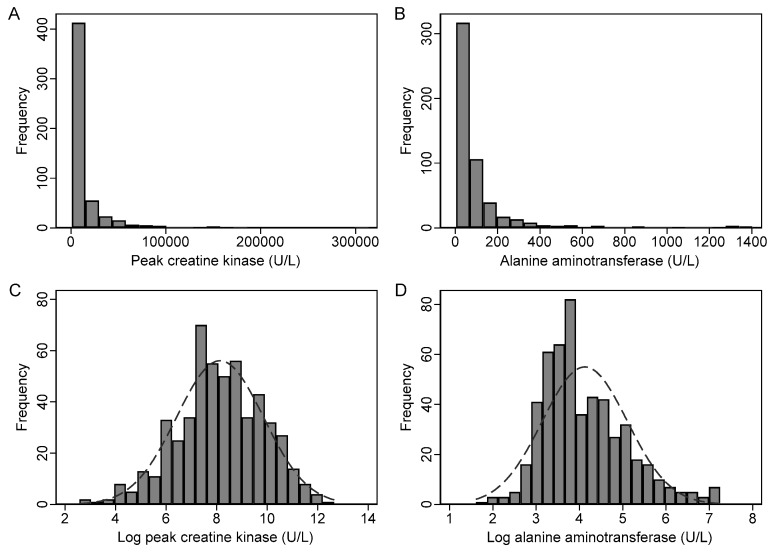
Effect of logarithmic transformation on the distribution of peak CK and ALT. Histograms demonstrate the distribution of the raw peak CK (**A**) and raw ALT (**B**), and the distribution after logarithmic transformation with a superimposed normal distribution curve for CK (**C**) and ALT (**D**).

**Figure 3:**
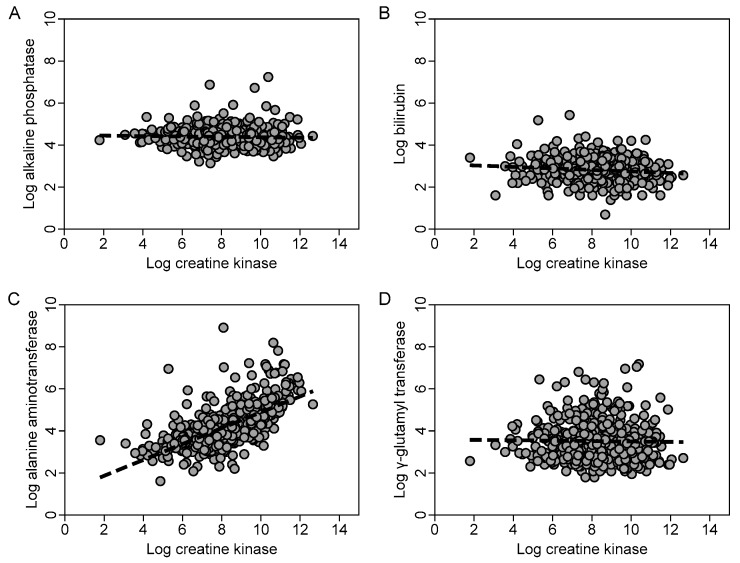
Scatterplots of log-transformed liver biochemistry and log-transformed peak creatine kinase (CK). The scatterplots (with line of best fit for linear regression) demonstrate a linear association between log CK and log-alanine aminotransferase (**C**) which was not evident with log-bilirubin (**B**), log alkaline phosphatase (**A**), and log γ-glutamyl transferase (**D**).

Due to the highly skewed distributions, log transformation was performed on the raw data prior to regression. For example, the distributions of the original data and log-transformed peak CK (log pCK) and log-transformed ALT (log ALT) are shown in Figure 2. The scatterplots (Figure 3) showed a positive linear relationship between log pCK and log ALT, and there was a moderate-strong linear correlation between these variables (*r* = 0.66, *p* < 0.001). However, there was only a weak or negligible linear correlation between log pCK and log-transformed bilirubin (*r* = −0.12, *p* = 0.004), log-transformed ALP (*r* = −0.07, *p* = 0.10), and log-transformed GGT (*r* = −0.01, *p* = 0.82), respectively.

### 3.5. Linear Regression

In the univariable analysis, the factors associated with the log ALT are summarized in Table 4. A statistically significant positive association was noted for log pCK, chronic liver disease, illicit drug use, diabetes, and AKI stage. A statistically significant negative association was noted for age, diabetes, CKD stage, and the number of PHMs. The variables with the highest coefficient of determination (*R*^2^) were log pCK, age, and AKI stage.

#### 3.5.1. Potential Confounders

There was a moderate linear relationship between log pCK and age (*r* = −0.35, *p* < 0.001), and between log pCK and AKI stage (*r* = 0.30, *p* < 0.001). There was a weak linear relationship between log pCK and CKD stage (*r* = −0.19, *p* < 0.001). For the categorical independent variables, log pCK showed an association with illicit drug use (*t* = 4.72, *p* < 0.001), chronic liver disease (*t* = 2.01, *p* = 0.044), cardiovascular disease (*t* = 3.42, *p* < 0.001), and diabetes (*t* = 3.43, *p* < 0.001). However, the number of PHMs used did not show any correlation with log pCK (*r* = 0.00, *p* = 0.98), even when analyzed categorically with ANOVA (*F*_3525_ = 2.39, *p* = 0.07).

#### 3.5.2. Multiple Regression

For the baseline multivariable model, we included log pCK, age, and AKI stage, which together accounted for most of the variance in log ALT. To the base model, we added variables identified in the univariable analysis with a *p* < 0.10, in order of the highest to lowest *R*^2^. Potential confounders that were not statistically significant and did not significantly affect the regression coefficient for log ALT were not included in the final multivariable model. Illicit drug use was not significant after adjusting for log pCK, age, and AKI stage (*b* = 0.06, *p* = 0.61) and only increased *b* (log ALT) by 0.4%. CKD stage was not significant after allowing for the other variables (*b* = −0.03, *p* = 0.54) and only increased *b* (log ALT) by 0.1%. Chronic liver disease was significant in the multivariable model and was added to the model. The number of PHMs was significant but destabilized the association between age and log ALT in the model (*b* = −0.03, *p* = 0.10), such that age and medications were not independently associated with log ALT. This is because older patients were more likely to receive more medications. On average, patients receiving one or more PHMs were 7.4 (95% CI: 3.8–11.0) years older than those not receiving any PHMs (*t* = 4.05, *p* < 0.001). As PHM use was not a potential confounder (no association with log pCK) or actual confounder (change in *b* (log ALT) of 1.1%), it was not included in the multivariable model. Diabetes status was not significant after allowing for the other variables (*b* = −0.02, *p* = 0.78) and only reduced *b* (log ALT) by 0.1%. Cardiovascular disease was not significant after allowing for the other variables (*b* = 0.08, *p* = 0.27) and only increased *b* (log ALT) by 0.1%. As a final check, variables previously eliminated were added back and the model was reanalyzed. There were no significant statistical interactions between the variables in the final model, which is shown in Table 5.

#### 3.5.3. Regression Diagnostics

In the multivariable model, the residuals were normally distributed (Supplementary Figure S1) and the linearity assumptions for age and AKI stage were confirmed using fractional polynomials. There was one potential outlier that did not affect the coefficient for log ALT (−1.3%) or model fit (*R*^2^ unchanged), so it was not excluded. There were no highly influential observations detected.

#### 3.5.4. Interpretation of Multiple Regression Model

**Figure 4:**
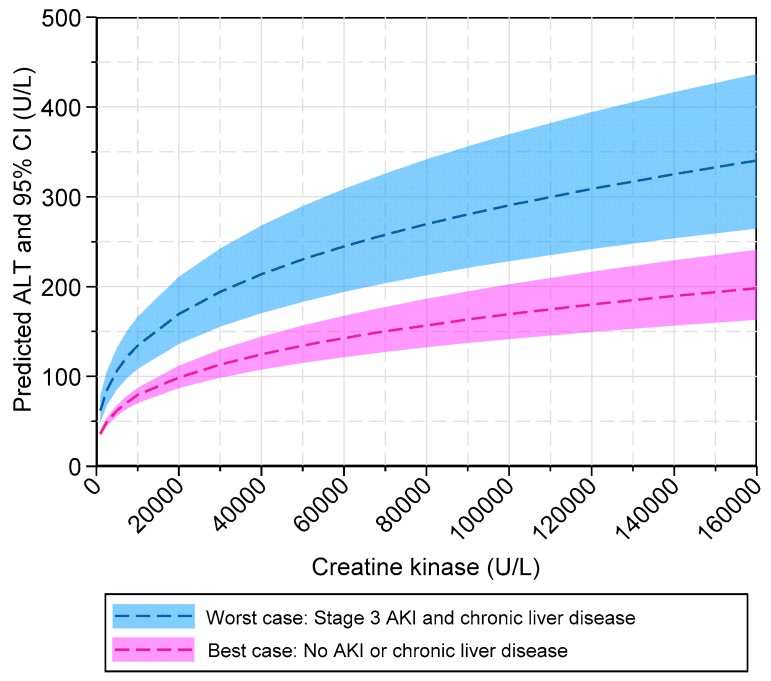
Model of predicted serum ALT in rhabdomyolysis. The multiple regression model was used to generate predicted levels of alanine aminotransferase (ALT) and associated 95% confidence intervals, based on peak creatine kinase, adjusted for acute kidney injury (AKI) stage and chronic liver disease status. Two prediction bands are represented, showing the best-case scenario (pink) and worst-case scenario (blue). The predictions were estimated while holding age constant at the group mean of 66 years.

The final multiple regression model explained 46% of the variance in log ALT. From the standardized coefficients (*β*), we showed that log pCK had a strong effect size on log ALT but AKI stage, age, and chronic liver disease only had a weak effect. The semi-partial *R*^2^ estimates indicated that age and chronic liver disease contributed less than 1% of unique information to explaining the variance in log ALT. Thus, a parsimonious model could just include log pCK and AKI stage as the independent variables. Given the log–log transformation of serum CK and ALT used for the regression model, the interpretation of the exponentiated coefficients needs to be specifically mentioned. On average, and holding the other variables constant, for every 10% increase in peak CK, there is a 3.2% increase in ALT. For every stage increase in AKI severity, there is a 12% increase in the geometric mean of ALT, after adjusting for the other variables. For every 20 years increase in age, there is a 4.9% decrease in the geometric mean of ALT, after adjusting for the other variables. Patients with chronic liver disease have a 24% increase in the geometric mean of ALT, after adjusting for the other variables. The relationship between peak CK and predicted ALT modeled by the regression equation is demonstrated in Figure 4.

### 3.6. Ratio of CK to ALT

The distribution of the CK/ALT ratios was highly skewed to the right, with a mean of 134 (SD, 152) and a median of 90 (IQR, 48–162). Using a non-parametric test based on ranks, we found no difference in the distribution of CK/ALT ratios by illicit drug use status (*p* = 0.11), use of PHMs (*p* = 0.13), or hepatitis virus replication (*p* = 0.14). On the other hand, the log pCK/log ALT ratios approximated a normal distribution and there was still no difference in the means of log pCK/log ALT by illicit drug use status (*t* = 0.23, *p* = 0.82) or hepatitis virus replication (*t* = 1.36, *p* = 0.18). However, there was a positive association between PHM use and the log pCK/log ALT ratio, with the ANOVA (*F*_3524_ = 5.6, *p* < 0.001) and Bonferroni’s multiple comparison tests indicating that patients receiving two or three PHMs had higher mean ratios than those receiving none.

### 3.7. Investigations for Abnormal Liver Function

The frequency of tests performed to investigate abnormal liver function and the proportion reported as abnormal are shown in Appendix B Table A3. There were 115 imaging studies performed in 96/528 patients, and 29% of ultrasonography, 48% of computed tomography scans, and 25% of magnetic resonance imaging scans performed did not detect abnormalities. The commonest abnormal finding was hepatic steatosis. Others included cirrhosis, metastatic disease, liver abscess, and hepatobiliary disease. Among the 92/528 patients who had additional blood workup (iron and copper studies, α1-antitrypsin levels, autoimmune studies, and hepatitis virus serology or polymerase chain reaction (PCR), the yield was low but the commonest finding was hepatitis C virus infection (17/92), followed by hepatitis B virus infection (2/92). Of the 19 hepatitis virus PCR positive cases, 14 (74%) were detected in patients with known chronic viral hepatitis, while five were new diagnoses. One case of suspected autoimmune disease was eventually diagnosed with paraneoplastic polymyositis. One patient had iron overload from recurrent transfusions but no patients with copper overload or α1-antitrypsin deficiency were identified.

### 3.8. Sensitivity Analysis

To account for possible liver injury, we excluded 73 patients with abnormal imaging (*n* = 59), active viral hepatitis detected by a quantitative PCR (*n* = 19), or iron overload (*n* = 1). In this select group of 455 patients, age (*b* = −0.05, *p* = 0.15) and chronic liver disease (*b* = −0.04, *p* = 0.74) were not significant. The log pCK maintained its strong association with log ALT (*b* = 0.36, 95% CI: 0.32–0.40, *p* < 0.001; *β* = 0.65), and AKI remained significant (*b* = 0.10, 95% CI: 0.04–0.16, *p* = 0.002; *β* = 0.11). The result of the reduced variance was a small increase in the *R*^2^ to 48%, compared to 46% of the original model. On average, for every 10% increase in peak CK, there was a 3.5% (95% CI: 3.1%–3.9%) increase in ALT after allowing for AKI stage.

### 3.9. Inpatient Mortality

The overall inpatient mortality was 8.9% (47/528). We found no difference in the means of log ALT between patients who died and the survivors (difference = 0.18, 95% CI: −0.48 to 0.11, *p* = 0.23). However, the mean log pCK/log ALT ratio was lower in patients who died compared to patients who survived (difference = 0.20, 95% CI: 0.08–0.31, *p* = 0.001). In the logistic regression analysis, log ALT was not associated with inpatient mortality. However, the pCK/ALT and log pCK/log ALT ratios were both associated with inpatient mortality. These results are summarized in Table 6.

## 4. Discussions

We examined the relationship between serum CK and liver function tests in patients with rhabdomyolysis. Older patients with pressure injuries comprised the majority of the study population. The range of CK and liver function tests were wide, with a significant positive skew in the distribution. Using multiple regression, we demonstrated a statistically significant positive linear relationship of medium to large effect between the log pCK and log ALT level. In contrast, the association between the log pCK and log-bilirubin, log ALP and log GGT were negligible.

Studies examining the nature of the relationship between CK and ALT in patients with rhabdomyolysis are limited. Experiments in healthy subjects with normal baseline liver function have proven that rhabdomyolysis can be associated with elevated aminotransferases [4,6,11]. These studies have focused on the temporal association rather than functional form, and thus not attempted to quantify this relationship. Weibrecht et al. reviewed 215 cases of rhabdomyolysis and examined the trajectory of the CK and aminotransferases (ALT and AST). Much like our data, the investigators noted that peak CK and ALT were not normally distributed. They used a stratified analysis and non-parametric tests to show differences in the ALT levels between the strata of peak CK levels [7]. In our study, we have maintained the interval level of measurements by regressing the log ALT on log pCK. Therefore, we could draw a quantitative inference from our analysis which, to our knowledge, has not been previously reported.

In our data, age showed a moderately strong negative linear relationship with log pCK, while AKI stage showed a moderately strong positive linear relationship with log pCK. In some studies, ALT declined with age. When age was analyzed categorically as an ordinal variable or in quartiles, serum ALT declined with increasing age while AST remained stable [19,20,21]. In studies using polynomial regression, the relationship between age and ALT is more complex. Elinav et al. studied subjects from family practice clinics and aged care homes and noted that a quadratic polynomial of age was a better fit than linear regression in modeling the relationship with ALT, resulting in an inverted parabolic relationship, with a peak ALT at 40–55 years. The authors also found no correlation between age and AST [22]. Vespasian-Gentilucci et al., reporting a large Italian study of over 44000 general practice subjects, noted a similar finding of an inverted parabolic relationship between age and ALT, with a peak in the third to fifth decade [23]. In our study, using a quadratic term did not improve the model fit when regressing log ALT on age.

Our finding of the association between AKI severity and ALT was interesting. In a study of nontraumatic rhabdomyolysis and reversible liver dysfunction, patients with AKI requiring dialysis had higher ALT levels than those who did not have AKI [24]. In critically ill patients, patients with an ALT or AST levels greater than 1000 U/L were more likely to have AKI than those with levels below 1000 U/L [13]. While there is some data to support our finding, it does raise the question of the mechanism involved. It is hypothesized that reno-hepatic crosstalk exists. In the setting of AKI, metabolic acidosis and numerous accumulated soluble mediators may promote inflammation and oxidative stress in the liver. Some of the toxins and modified proteins potentially involved include interleukin-1, interleukin-6, interleukin-18, tumor necrosis factor-α, asymmetrical dimethylarginine, urea, creatinine, and indoxyl sulfate [25]. Oxidative stress has been well described in AKI related to rhabdomyolysis [26], and a similar phenomenon may involve the liver. However, most data come from animal studies and validation in humans is required.

In the univariable analysis, CKD stage showed a negative linear relationship with log ALT. There have been other observational studies to support this finding. Fabrizi et al. noted that AST and ALT levels were lower in patients with pre-dialysis and dialysis-dependent CKD, compared to age-matched healthy controls without serological evidence of hepatitis virus infection [27]. A similar finding was noted by Ray et al., who also noted an ordinal relationship between ALT and CKD stage [28]. In the large Italian study of general practice patients, a higher serum creatinine was associated with a lower ALT [23]. The mechanism is unknown but may be related to pyridoxine deficiency [29] or inhibition of aminotransferase activity by uremic solutes [30]. In our study, the effect of CKD stage on log ALT was not independent of age and log pCK. This may be because the prevalence of CKD increases with age [31] and CKD is also associated with muscle atrophy [32]. Indeed, patients with reduced eGFRs had lower peak CKs (median, 3303 U/L (IQR, 1909–6910 U/L)) than those with normal eGFRs (median, 6855 U/L (2643–22,122 U/L)), which was statistically significant (*z* = 5.74, *p* < 0.001).

The final variable of significance was chronic liver disease, which is intuitive. However, the study by Jo et al. reported that serum aminotransferases were not significantly different between patients with chronic liver disease and those without, in the setting of rhabdomyolysis. Of the 19/165 (11.5%) of patients with chronic liver disease, 13/19 were due to alcoholic liver disease [33]. In our study, the proportion of patients with chronic liver disease was similar but there was a higher proportion of patients with chronic viral hepatitis. In our sensitivity analysis, we excluded patients with abnormal imaging or active hepatitis virus replication. When these patients were removed, chronic liver disease was not significant in the multivariable model. However, we felt it was best to avoid excluding patients so that the model can be applied to a wide range of settings. Furthermore, chronic liver disease only uniquely explained less than 1% of the variance in log ALT, so the risk of bias was negligible.

We found no association between sex and log ALT. This contrasts with other studies that reported lower baseline ALT levels in females [22,23]. It is possible that the small sex-related differences in normal subjects did not translate to a detectable difference in pathological states. Other studies have also suggested that overweight and obesity are associated with elevated aminotransferases, using body mass index and other metabolic markers to correlate with ALT [23,34,35]. However, we were not able to address this due to missing data on body mass index. In our univariable analysis, diabetes status showed a negative correlation with log ALT. In most epidemiological studies, diabetes and pre-diabetic states are associated with higher baseline ALT levels [36,37]. The link between diabetes and elevated baseline ALT is usually non-alcoholic fatty liver disease [38]. We believe our findings could be explained by the lower peak CK levels in diabetics (median, 3303 U/L (IQR, 1762–6899 U/L)) compared to non-diabetics (median, 4549 U/L (IQR, 2467–16694 U/L)), which was statistically significant (*z* = 3.53, *p* < 0.001). There is evidence to link diabetes with muscle atrophy [39], which most likely explains this finding. The prevalence of diabetes is also increased with age, and we have already shown that ALT levels are negatively correlated with age. Thus, it was likely that the effect of diabetes on ALT was mostly explained by log pCK and age in the multivariable model. Similarly, patients who used illicit drugs were younger and had higher peak CKs [15], so that age and log pCK may have already accounted for most of the variance in log ALT due to illicit drug use status in the multivariable model.

Some studies have advocated using the CK/ALT ratio to help distinguish genuine liver injury from isolated ALT release from muscle [40,41]. In theory, a small ratio suggests underlying liver injury, but a definitive cut-off is not established. In our study, this ratio may be relevant in patients using illicit drugs or PHMs, given the potential for drug-induced liver injury, and those with active hepatitis virus replication. We found that the CK/ALT ratio and log pCK/log ALT ratio provided slightly different results, with the latter confirming the negative association between PHM use and log ALT in the univariable regression, and the former did not. The reason for the negative correlation between PHM and log ALT levels was unclear to us but at least it does suggest that it was highly unlikely that the use of PHMs, as defined in this study, contributed to an elevated ALT.

We did not find an association between serum ALT and inpatient mortality as previously suggested in a study of critically ill patients [13]. On the other hand, a higher ratio of CK to ALT, both as raw values and log-transformed values, was associated with a lower odds of inpatient mortality, even after adjusting for age, AKI and sepsis. For example, a one-unit increase in the log pCK/log ALT ratio was associated with a 71% lower odds of inpatient death, on average, after allowing for age, AKI, and sepsis. This finding supports the idea that a muscle source of elevated ALT may be more benign compared to liver-derived ALT elevations. Thus, the CK/ALT ratio may have some usefulness as a prognostic marker which deserves further evaluation in patients with severe rhabdomyolysis.

### 4.1. Strengths and Limitations

To our knowledge, this is the first study to model the relationship between peak CK and ALT in rhabdomyolysis in a diverse group of patients and provide a quantitative estimate for inference. The other strength of the study was the identification and analysis of most of the variables known or suspected to confound the association between CK and ALT. The limitations include the retrospective design and lack of data on AST, which was not routinely tested in our network. The baseline liver function was not available for all patients, so that an elevated ALT may be a pre-existing problem in some patients. However, including chronic liver disease into the multivariable model should minimize this bias. We also showed some evidence that the abnormal ALT was transient in many cases, and would normalize prior to discharge. However, this data was also limited by the availability of biochemistry and the timing of discharge, and we had no histological data from liver biopsy. Finally, we also conducted a sensitivity analysis by excluding patients with active hepatitis virus replication or imaging abnormalities and showed that the change in the coefficient for log pCK was minimally affected. With regard to the possibility of undiagnosed ischemic hepatitis, we attempted to account for this by including a sepsis variable, the most common cause of shock and ischemic hepatitis. Finally, the timing of peak ALT may be different to CK, which may occur 24–48 h after CK as demonstrated by experimental, exercise-induced rhabdomyolysis in humans [6], so that the predicted ALT may also have been underestimated.

#### Model Assumptions

We made the assumption that no other potential sources of ALT besides skeletal muscle and liver (e.g., unrecognized myocardial injury) contributed to the elevated ALT in patients with rhabdomyolysis. We also assumed that no specific treatment (e.g., fluid resuscitation) confounded the association between log pCK and ALT. Currently, it is unclear if fluid resuscitation can affect ALT levels independent of CK. As treatment effect could not be reliably quantified in our study and tested in our regression models, this remains a possibility that may have biased our estimates. However, if the endpoint of successful fluid resuscitation is prevention of AKI, then this covariate is already included in the multivariable analysis and could have accounted for treatment effect.

### 4.2. Implications for Clinical Practice

By determining the functional form of the relationship between peak CK and ALT, the peak CK can be used to anticipate the rise in ALT with rhabdomyolysis. If the ALT falls outside the upper 95% confidence interval of the predicted ALT for a given peak CK level, age, and chronic liver disease status, it would raise suspicion of concurrent liver injury. For example, we would expect that a 66-year-old patient with a peak CK of 100,000 U/L without AKI or chronic liver disease to have less than a 5% probability of recording an ALT above 200 U/L due to isolated muscle injury. Given that there were few patients with peak CKs above 160,000 U/L, we do not recommend extrapolating above this level. We also confirmed that on average, there is no clinically detectable change in serum bilirubin, ALP, and GGT in rhabdomyolysis. Thus, significant increases in these markers are altogether additional evidence of liver injury. 

### 4.3. Research Recommendations

We hope our findings will be validated in future studies, and suggest others consider examining the log CK/log ALT ratio in rhabdomyolysis. We suspect that novel biomarkers will have a role in the future in helping differentiate liver injury from muscle injury without solely relying on ALT levels [42], which we have shown to lack specificity.

## 5. Conclusions

An isolated increase in serum ALT can occur with rhabdomyolysis. Peak CK levels, as a marker of severity of muscle injury, is the single most important variable explaining the increased ALT. Age, AKI, and chronic liver disease were minor contributors but together these four variables explained half of the variance in ALT. It may be possible to anticipate the rise in ALT based on the peak CK.

## Figures and Tables

**Table 1:** Characteristics of included patients (*n* = 528).

Characteristic	*n* (%)
Median age (IQR ^1^), years	74 (52–84)
Male sex	291 (55.1)
Diabetes	123 (23.3)
Cardiovascular disease ^2^	171 (32.4)
Active cancer	32 (6.1)
Estimated GFR (mL/min/1.73 m^2^):	
Above 89 (CKD ^3^ stage 0 and 1)	241 (45.6)
60 to 89 (CKD stage 2)	181 (34.3)
30 to 59 (CKD stage 3)	94 (17.8)
Below 30 (CKD stage 4 and 5)	12 (2.3)
Acute kidney injury:	
None	252 (47.7)
Stage 1	124 (23.5)
Stage 2	63 (11.9)
Stage 3	89 (16.9)
Sepsis	84 (15.9)
Illicit drug use	69 (13.1)
Chronic liver disease	62 (11.7)
History of excessive alcohol intake	100 (18.9)
Use of potentially hepatotoxic medications	311 (58.9)
Statin treatment ^4^	188 (35.6)

^1^ Interquartile range; ^2^ Composite of coronary artery disease, peripheral vascular disease, and stroke; ^3^ Chronic kidney disease; ^4^ Subgroup of potentially hepatotoxic medications.

**Table 2:** Serum biochemistry at peak creatine kinase (CK) level showing median and interquartile range.

Variable	Baseline ^1^	Admission	Peak
Albumin, g/L	35 (31–39)	34 (30–38)	33 (28–37)
Bilirubin, µmol/L	12 (8–17)	16 (11–23)	16 (11–22)
Alkaline phosphatase, U/L	83 (67–102)	83 (65–107)	78 (61–102)
Alanine transaminase, U/L	23 (15–36)	45 (28–95)	48 (31–108)
γ-glutamyl transferase, U/L	30 (20–61)	30 (18–57)	30 (17–58)
International normalized ratio	-	1.2 (1.1–1.4) ^2^	1.2 (1.1–1.4) ^2^
Creatine kinase, U/L	-	3241 (1542–8710)	4238 (2224–13,044)
Creatine kinase/ALT ratio	-	76 (41–140)	90 (49–162)

^1^ Data not available in 402/528 (76.1%) of patients; ^2^ data missing in 246/528 (46.6%) on admission and 231/528 (43.8%) at peak CK, and also included patients on warfarin anticoagulation.

**Table 3:** Cross-tabulation of alanine aminotransferase (ALT) at peak CK and at time of discharge.

Abnormal ALT at Peak CK	Abnormal ALT at Discharge		Total
	No	Yes	
No	49	15	64
Yes	46	119	165
Total	95	134	229

**Table 4:** Univariable linear regression analysis.

Variable	Coef. (95% C.I.)	*p*-Value	*R*^2^ (%) ^2^
Age, per 20-year increase	−0.33 (−0.41, −0.25)	<0.001	12.1
Female sex	0.01 (−0.16, 0.19)	0.86	0.0
Diabetes	−0.28 (−0.48, −0.08)	0.006	1.4
Cardiovascular disease ^1^	−0.17 (−0.36, 0.00)	0.053	0.7
Active cancer	−0.02 (−0.37, 0.34)	0.91	0.0
Chronic liver disease	0.46 (0.20, 0.73)	0.001	2.2
History of alcohol excess	0.10 (−0.11, 0.32)	0.34	0.2
Number of hepatotoxic medications	−0.15 (−0.25, −0.05)	0.004	1.6
Illicit drug use	0.61 (0.36, 0.86)	<0.001	4.3
Chronic kidney disease, per stage	−0.22 (−0.32, −0.11)	<0.001	3.2
Acute kidney injury, per stage	0.28 (0.21, 0.35)	<0.001	10.2
Log-transformed peak creatine kinase	0.38 (0.34, 0.42)	<0.001	42.9
Sepsis	0.18 (−0.06, 0.41)	0.14	0.4

^1^ Composite of stroke, peripheral vascular disease, and ischemic heart disease; ^2^ Coefficient of determination.

**Table 5:** Multivariable linear regression model for log ALT (*n* = 528).

Variable	*b* (95% C.I.) ^1^	*p*-Value	*β* ^2^	Semipartial *R^2^* (%) ^3^
Log peak CK	0.33 (0.29, 0.38)	<0.001	0.58	26.0
AKI stage	0.11 (0.05, 0.17)	<0.001	0.12	1.37
Age per 20 years	−0.07 (−0.14, −0.01)	0.034	−0.08	0.47
Chronic liver disease	0.21 (0.01, 0.41)	0.039	0.07	0.44

^1^ Unstandardized coefficients; ^2^ standardized coefficients; ^3^ squared semipartial correlation.

**Table 6:** Logistic regression of inpatient mortality on alanine aminotransferase (ALT) variables (*n* = 528).

Variable	Odds Ratio ^1^	95% C.I.	*p*-Value
Log ALT	1.24	0.88–1.75	0.21
pCK^2^/ALT per 10-unit increase	0.92	0.87–0.97	0.002
Log pCK/log ALT	0.29	0.12–0.67	0.004

^1^ Adjusted for age, acute kidney injury stage, and sepsis; ^2^ peak creatine kinase.

## Supplementary Materials

The following are available online at https://www.mdpi.com/2077-0383/9/1/81/s1, Table S1: Categories of potentially hepatotoxic medications used prior to peak creatine kinase; Figure S1: Regression diagnostics for final model.

## Appendix A

**Table A1:** Implicated causes of rhabdomyolysis.

Category ^1.^	*n* (% total)
Pressure injury	380 (72.0)
Drugs and toxins	90 (17.1)
Trauma	41 (7.8)
Infection	32 (6.1)
Exertional	27 (5.1)
Seizures	23 (4.4)
Acute limb ischemia	16 (3.0)
Operative	15 (2.8)
Electrolyte disorder	11 (2.1)
Thermal extremes	9 (1.7)
Myositis	3 (0.6)
Inherited disease	2 (0.4)

^1^ More than one cause may be implicated in a patient.

**Table A2:** Categories of illicit drug use.

Category ^1^	*n* (% total)
Benzodiazepines	19 (3.6)
Cannabinoids	31 (5.9)
Cocaine	3 (0.6)
Meth/amphetamines	35 (6.6)
Opioids	34 (6.4)
Other ^2^	7 (1.3)

^1^ Categories are not mutually exclusive; ^2^ includes ecstasy, γ-hydroxybutyrate, lysergic acid diethylamide, and N-methyl-D-aspartate receptor antagonist.

## Appendix B

**Table A3:** Diagnostic tests to investigate abnormal liver function tests.

Diagnostic Tests Performed ^1^	Tested *n* (% total)	Abnormal *n* (% tested)
Hepatitis serology or PCR	74 (14.0)	19 (25.7)
Iron studies	19 (3.6)	1 (5.3)
Copper studies	13 (2.5)	0 (0)
Autoimmune studies	47 (8.9)	1 (2.1) ^2^
Alpha1-antitrypsin	6 (1.1)	0 (0)
Liver ultrasound	65 (12.3)	46 (70.8)
Liver CT	46 (8.7)	24 (52.2)
Liver MRI	4 (0.8)	3 (75.0)

^1^ Tests were not mutually exclusive; ^2^ paraneoplastic polymyositis.
