# Supplementary material for: A Cross-Sectional Study of the Relationship between Serum Creatine Kinase and Liver Biochemistry in Patients with Rhabdomyolysis
Source: J Clin Med. 2019 Dec 28;9(1):81. doi: 10.3390/jcm9010081 (PMC7019809; doi:10.3390/jcm9010081)
Supplement: Supplementary file 1 [file jcm-09-00081-s001.zip › Supplementary Table S1.pdf]

Table S1. Categories of potentially hepatotoxic medications used prior to peak creatine kinase.

**Categories** [1]	***n* (% total)**
**1. Psychotropics**	
Quetiapine	36 (6.8)
Mirtazapine	20 (3.8)
Olanzapine	10 (1.9)
Amitriptyline	9 (1.7)
Others	12 (2.3)
**2. Antibiotics**	
Ceftriaxone	8 (1.5)
Cephalexin/cephazolin	4 (0.8)
Flucloxacillin	3 (0.6)
Azithromycin	3 (0.6)
Others	6 (1.1)
**3. Antiepileptics**	
Sodium valproate	16 (3.0)
Carbamazepine	13 (2.5)
Lamotrigine	3 (0.6)
**4. Lipid lowering**	
Statin	188 (35.6)
Fenofibrate	1 (0.2)
**5. Rheumatological**	
Allopurinol	17 (3.2)
Methotrexate	8 (1.5)
Leflunomide	4 (0.8)
Azathioprine	2 (0.4)
**6. Cardiovascular**	
Nifedipine	3 (0.6)
Amiodarone	2 (0.4)
Verapamil	2 (0.4)
**7. Analgesic**	
Acetaminophen*	75 (14.2)
**8.Miscellaneous**	
Others	4 (0.8)

[1] only the top four medications of each category are shown and medication use was not mutually exclusive;
[2] one overdose excluded
