# Supplementary figures and images for: A Cross-Sectional Study of the Relationship between Serum Creatine Kinase and Liver Biochemistry in Patients with Rhabdomyolysis
Source: J Clin Med. 2019 Dec 28;9(1):81. doi: 10.3390/jcm9010081 (PMC7019809; doi:10.3390/jcm9010081)

**A**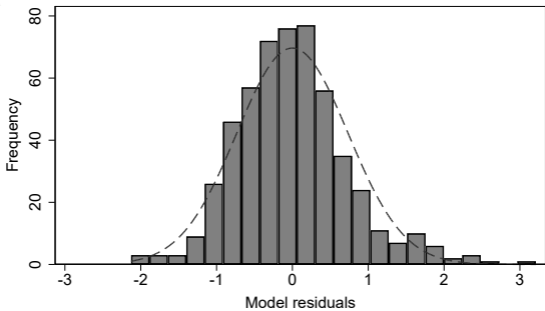**B**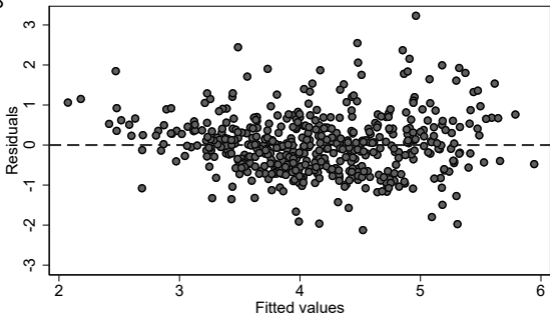

Supplement: Supplementary file 1 [file jcm-09-00081-s001.zip › Supplementary Figure S1.pdf]
